# Clinical characterization and outcomes of impulse oscillometry-defined bronchodilator response: an ECOPD cohort-based study

**DOI:** 10.1186/s12931-024-02765-7

**Published:** 2024-03-30

**Authors:** Lifei Lu, Fan Wu, Jieqi Peng, Xiaohui Wu, Xiangqing Hou, Youlan Zheng, Huajing Yang, Zhishan Deng, Cuiqiong Dai, Ningning Zhao, Kunning Zhou, Qi Wan, Gaoying Tang, Jiangyu Cui, Shuqing Yu, Xiangwen Luo, Changli Yang, Shengtang Chen, Pixin Ran, Yumin Zhou

**Affiliations:** 1 State Key Laboratory of Respiratory Disease, National Clinical Research Center for Respiratory Disease, National Center for Respiratory Medicine, Guangzhou Institute of Respiratory Health, The First Affiliated Hospital of Guangzhou Medical University, Guangzhou, China; 2 Guangzhou National Laboratory, Guangzhou, China; 3 Lianping County People’s Hospital, Heyuan, China; 4 Wengyuan County People’s Hospital, Shaoguan, China

**Keywords:** Impulse oscillometry, Bronchodilator response, Small airway dysfunction, Decline in lung function, Acute exacerbations

## Abstract

**Background:**

The clinical significance of the impulse oscillometry-defined small airway bronchodilator response (IOS-BDR) is not well-known. Accordingly, this study investigated the clinical characteristics of IOS-BDR and explored the association between lung function decline, acute respiratory exacerbations, and IOS-BDR.

**Methods:**

Participants were recruited from an Early Chronic Obstructive Pulmonary Disease (ECOPD) cohort subset and were followed up for two years with visits at baseline, 12 months, and 24 months. Chronic obstructive pulmonary disease (COPD) was defined as a post-bronchodilator forced expiratory volume in 1 s (FEV_1_)/forced vital capacity (FVC) ratio < 0.70. IOS-BDR was defined as meeting any one of the following criteria: an absolute change in respiratory system resistance at 5 Hz ≤ − 0.137 kPa/L/s, an absolute change in respiratory system reactance at 5 Hz ≥ 0.055 kPa/L/s, or an absolute change in reactance area ≤ − 0.390 kPa/L. The association between IOS-BDR and a decline in lung function was explored with linear mixed-effects model. The association between IOS-BDR and the risk of acute respiratory exacerbations at the two-year follow-up was analyzed with the logistic regression model.

**Results:**

This study involved 466 participants (92 participants with IOS-BDR and 374 participants without IOS-BDR). Participants with IOS-BDR had higher COPD assessment test and modified Medical Research Council dyspnea scale scores, more severe emphysema, air trapping, and rapid decline in FVC than those without IOS-BDR over 2-year follow-up. IOS-BDR was not associated with the risk of acute respiratory exacerbations at the 2-year follow-up.

**Conclusions:**

The participants with IOS-BDR had more respiratory symptoms, radiographic structural changes, and had an increase in decline in lung function than those without IOS-BDR.

**Trial registration:**

Chinese Clinical Trial Registry, ChiCTR1900024643. Registered on 19 July, 2019.

**Supplementary Information:**

The online version contains supplementary material available at 10.1186/s12931-024-02765-7.

## Introduction

Airflow limitation responsiveness is assessed with bronchodilator response (BDR) testing, which is a diagnostic tool for asthma [1, 2]. BDR is commonly evaluated using spirometry and is known as spirometric BDR [3–5]. Previous studies revealed that 18.4–52.7% of participants with chronic obstructive pulmonary disease (COPD) exhibited spirometric BDR [6–8]. However, the clinical significance of spirometric BDR in patients with COPD remains controversial. Numerous studies have reported no association between spirometric BDR and exacerbations, mortality, or hospitalization rates in patients with COPD after adjusting for baseline function [6, 7, 9], however, a few studies have presented contrary conclusions [8, 10].

Currently, spirometric BDR testing primarily reflects large airway obstruction responsiveness and correlates poorly with clinical symptoms [11]. Small airways are the predominant obstruction sites in COPD [12, 13]. Nevertheless, the clinical significance of BDR in small airways in COPD is uncertain. Therefore, there is an urgent need for new tools to evaluate small airway BDR.

Impulse oscillometry (IOS) is more sensitive for detecting peripheral airways and small airway BDR changes than spirometry [14–18]. Since European Respiratory Society (ERS) guideline proposed a threshold for assessing small airway BDR using oscillation in healthy participants [19, 20], several studies have explored different thresholds for BDR testing in small airways using IOS (IOS-BDR) [20–22]. In patients with COPD, many studies have only reported changes in IOS parameters after bronchodilator use or distinguished between asthma and COPD using IOS [23–25]. However, to the authors’ best knowledge, very few studies reported on clinical characterization and longitudinal prognosis of IOS-BDR using a fixed threshold. Alice M et al. had found that oscillation parameters were more sensitive in identifying poor asthma control than spirometry [14]. Henrik’s study showed that abnormal response in oscillation parameters had a higher prevalence of asthma and wheeze compared with participants with a normal response to bronchodilation [21]. These study more forced on effect of oscillation on symptoms and asthma control in patients with asthma, but the clinical significance of the IOS-BDR in COPD was not well-known. BDR is recognized as a “treatable traits” of COPD. Accordingly, identifying IOS-BDR clinical features would aid the formulation of a theoretical basis for COPD treatment.

Therefore, this study aimed to report clinical characteristics of IOS-BDR and the association between imaging changes, acute respiratory exacerbations, and lung function decline with IOS-BDR in participants through a prospective cohort study.

## Materials and methods

### Study participants

The Early Chronic Obstructive Pulmonary Disease (ECOPD) cohort is a prospective observational study aimed at investigating COPD early occurrence and development (Chinese Clinical Trial Registry ChiCTR1900024643). The cohort rationale and design have been previously reported [26]. From July 2020 to December 2021, a subset of individuals aged 40–80 years from the ECOPD cohort was continuously recruited from the community in this study. These participants included participants with spirometry-defined COPD [post-bronchodilator FEV_1_/forced vital capacity [FVC] ratio < 0.70] and participants without spirometry-defined COPD [post-bronchodilator FEV_1_/FVC ratio ≥ 0.70]. The participants were followed up for two years with visits at baseline, 12 months, and 24 months.

The participants completed the questionnaires and underwent pre-bronchodilator IOS tests, pre-bronchodilator spirometry tests, post-bronchodilator IOS tests, and post-bronchodilator spirometry tests. Participants were excluded if they met any of the following criteria at baseline: (1) age < 40 years or > 80 years; (2) incomplete spirometry tests or IOS tests; (3) respiratory infection or exacerbations within four weeks prior to screening; (4) heart attack (myocardial infarction and malignant arrhythmia) in the past three months. The previous cohort design report contains more details [26].

This study adhered to the ethical guidelines outlined in the Declaration of Helsinki. The research protocol was approved by the First Affiliated Hospital of Guangzhou Medical University Ethics Committee (Approval No. 2018-53) prior to study initiation. Written informed consent was obtained from all participants prior to their enrollment in the study.

### Questionnaire

The questionnaire in this study was revised in accordance with the Chinese COPD epidemiology study, including smoking status, pack-years, history of occupational exposure, family history of respiratory diseases, and history of asthma [27, 28]. Biomass exposure was defined as cooking or heating using biomass (mainly wood, crop residues, charcoal, grass, and dung) for more than 1 year. History of occupational exposure to dust/gases/fumes was defined as having occupational exposure to dust/gases/fumes for more than 1 year over a participants’ lifetime. We defined family history of respiratory diseases as having parents, siblings, and children with respiratory diseases (chronic bronchitis, emphysema, asthma, COPD, cor pulmonale, bronchiectasis, lung cancer, interstitial lung disease, obstructive sleep apnea hypopnea syndrome). Current asthma was defined as self-reported physician diagnosed asthma in combination with current use of asthmatic medication and/or asthma attack within the last 12 months and as self-reported physician diagnosed asthma in combination with the participant reporting to still having asthma. The degree of dyspnea and the participants’ health status were assessed using modified Medical Research Council dyspnea scale (mMRC) scores and COPD assessment test (CAT) scores, respectively [29]. Acute respiratory exacerbation events/exacerbations of COPD were specifically characterized by the onset or aggravation of at least two of the following five symptoms: cough, sputum, purulent sputum, dyspnea, and wheeze > 2 days after excluding other diseases. Moderate and severe acute respiratory exacerbations were characterized based on symptom worsening requiring treatment with antibiotics and/or systemic corticosteroids or treatment in a clinic, emergency department, or hospital setting. Acute exacerbation events/exacerbations of COPD can be classified as mild, moderate, and severe. The severity of acute respiratory exacerbations was assessed and recorded by well-trained staff according to the following categories: mild exacerbations were defined as those resulting in domiciliary management with COPD medications alone. Moderate exacerbations were defined as those resulting in outpatient or emergency department visits and the need for COPD medication. Severe exacerbations were defined as those resulting in hospitalization [30, 31].

### Computed tomography (CT)

Quantitative CT image assessment was conducted using multidetector-row CT scanners (Siemens Definition AS Plus 128-slicers and United Imaging uCT 760 128-slicers) combined with 3D Slicer 4.11 software on Chest Imaging Platform [26]. Emphysema was quantified by measuring each patient’s emphysema index, which was defined as the percentage of low-attenuation areas below − 950 Hounsfield units (HU) on full-inspiration CT. Air trapping was defined as the percentage of low-attenuation areas below − 856 HU on full-expiration CT [32].

### Spirometry

In accordance with ERS/American Thoracic Society (ATS) standards [33], the operator performed a 3-L volume spirometry calibration daily. The participants were instructed not to inhale any bronchodilator for at least 12 h and to avoid swallowing or air leakage during the operation and were required to complete at least three forced expiratory maneuvers until the largest and second-largest FEV_1_ and FVC values were within 150 mL. BDR was tested after a 20-min administration of 400 µg salbutamol through a 500-mL spacer.

### IOS

The mechanical properties of the respiratory system were measured using IOS [34]. Participants need breath lasting for more than 30 s and to avoid coughing, swallowing, and air leakage during tidal breathing [34]. The IOS parameters included respiratory system resistance at 5 Hz (R5), respiratory system resistance at 20 Hz (R20), the difference between R5 and R20 (R5-R20), respiratory system reactance at 5 Hz (X5), reactance area (AX), and resonant frequency (Fres). The absolute change was expressed as post-bronchodilator value minus pre-bronchodilator value, and IOS-BDR was defined as meeting any of the following criteria: absolute change in R5 ≤ − 0.137 kPa/L/s, absolute change in X5 ≥ 0.055 kPa/L/s, or absolute change in AX ≤ − 0.390 kPa/L [14, 19].

### Statistical analysis

Continuous variables with normal distribution are reported as the mean (standard deviation [SD]). Continuous variables that did not exhibit normal distribution are presented as the median [interquartile range (IQR)]. The differences in clinical characterization between participants with and without IOS-BDR were compared using Student’s t-test, the Wilcoxon rank-sum test, Fisher’s exact or chi-squared test. The difference between participants with and without IOS-BDR in terms of symptom scores (CAT scores), emphysema, and air trapping were examined with multivariable linear regression. The potential confounders considered were as follows: age, sex, body mass index (BMI), pack–years, smoking status, family history of respiratory diseases, occupational exposure, biomass exposure, and history of asthma. Associations between IOS-BDR and decline in lung function (FEV_1_, FVC, and FEV_1_/FVC ratio) were assessed using linear mixed-effects models, providing the mean change in lung function [35]. Baseline lung function was additionally included for confounding factor adjustment to analyze the rate of lung function decline. Baseline FEV_1_ and past exacerbation history were the most important risk factors for acute respiratory exacerbations. Thus, logistic regression modeling was used to evaluate associations between acute respiratory exacerbations outcomes within 2-year follow-up and IOS-BDR. Exacerbations were modeled as a binary outcome (0 vs. ≥ 1 episode) in the aforementioned logistic models adjusting for the potential confounders (age, sex, BMI, pack–years, smoking status, family history of respiratory diseases, occupational exposure, biomass exposure, and history of asthma), exacerbations in the previous year, and baseline pre-bronchodilator FEV_1_.

Subsequently, subgroup analyses were conducted, where the participants were stratified by sex, smoking status, and COPD. All statistical analyses were conducted using IBM SPSS 27.0 and SAS 9.4 (SAS Institute, Inc.), and a P-value less than 0.05 was considered statistically significant.

## Results

### Baseline characteristics

**Fig. 1:**
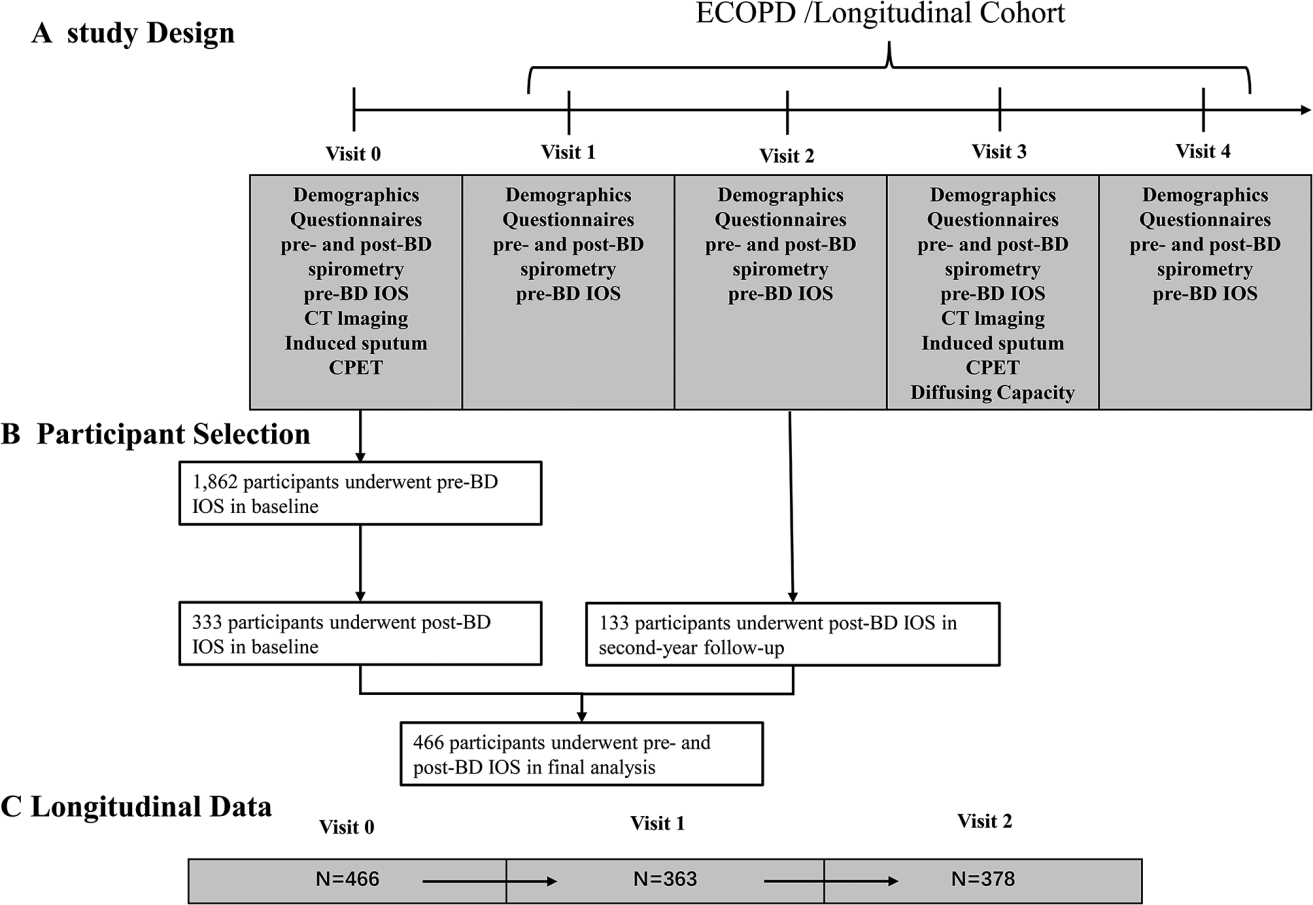
Study flow chart. Abbreviations: ECOPD = Early Chronic Obstructive Pulmonary Disease; IOS, Impulse oscillometry; CT, computed tomography; CPET, cardiopulmonary exercise testing. BD, bronchodilator

Figure 1 presents the inclusion and exclusion criteria for this study. Initially, 1862 participants completed pre-bronchodilator IOS tests at baseline in ECOPD cohort from July 2019 to August 2021, then only 466 participants underwent post-bronchodilator IOS tests. The participants in the present study were based on two parts: 333 participants underwent pre- and post-bronchodilator IOS tests in baseline from July 2020 to August 2021, and 133 participants underwent pre- and post-bronchodilator IOS tests in second-year followed-up from November 2021 to December 2021. Consequently, a final cohort of 466 participants was included for data analysis (92 participantss with IOS-BDR and 374 participants without IOS-BDR). These participants have completed a 2-year follow-up until December 2023. At baseline, the mean age of the total participants was 62.3 years (SD 8.0), 79.8% of the participants were males, and about 50% of the participants were current smokers. Compared with the participants without IOS-BDR, the participants with IOS-BDR had more chronic respiratory symptoms, such as cough (37.0% vs. 25.5%), wheeze (22.8% vs. 10.7%), and history of asthma (4.4% vs. 0.8%) (Table 1). Furthermore, the participants with IOS-BDR had more impaired lung function, more severe airflow limitation, higher airway resistance, and higher absolute change in IOS parameters than those without IOS-BDR (Table 2).

**Table 1:** Baseline characteristics of participants with and without IOS-BDR in overall participant

	Overall participants (*n* = 466)	Participants without IOS-BDR (*n* = 374)	Participants with IOS-BDR (*n* = 92)	*P*-Value
Age, years	62.3 ± 8.0	62.3 ± 8.0	62.2 ± 7.9	0.923
Male. *n* (%)	372 (79.8)	300 (80.2)	72 (78.3)	0.676
BMI, kg/m^2^	22.4 ± 3.2	22.4 ± 3.2	22.5 ± 3.3	0.830
Pack-years	30.4 ± 32.9	30.2 ± 32.6	31.5 ± 34.1	0.733
*Smoking status, * *n* * (%)*				0.757
Never smoker	129 (27.7)	104 (27.8)	25 (27.2)	
Ever smoker	103 (22.1)	85 (22.7)	18 (19.6)	
Current smoker	234 (50.2)	185 (49.5)	49 (53.3)	
*Chronic respiratory symptoms, * *n * *(%)*				
Cough	129 (27.7)	95 (25.5)	34 (37.0)	**0.028**
Phlegm	179 (38.5)	136 (36.5)	43 (46.7)	0.070
Wheeze	61 (13.1)	40 (10.7)	21 (22.8)	**0.002**
Dyspnea	153 (32.9)	116 (31.1)	37 (40.2)	0.095
Family history of respiratory diseases, *n* (%)	67 (14.4)	52 (13.9)	15 (16.3)	0.557
occupational exposure, *n* (%)	77 (16.5)	61 (16.3)	16 (17.4)	0.802
Biomass exposure, *n* (%)	133 (28.5)	103 (27.5)	30 (32.6)	0.335
History of asthma, *n* (%)	7 (1.5)	3 (0.8)	4 (4.4)	**0.041**
mMRC scores	0.4 ± 0.6	0.4 ± 0.6	0.5 ± 0.7	**0.047**
CAT scores	2.8 ± 3.4	2.5 ± 3.0	3.9 ± 4.5	**0.009**
LAA_− 950_	2.6 ± 4.5	2.4 ± 4.3	4.0 ± 5.1	**0.038**
LAA_− 856_	15.4 ± 17.2	14.2 ± 16.4	22.4 ± 20.2	**0.008**
Exacerbations in previous year*	0.35 ± 0.84	0.33 ± 0.84	0.44 ± 0.83	0.338
Follow-up, month	23.5 ± 0.7	23.5 ± 0.7	23.4 ± 0.6	0.262

Data are mean (standard deviation) or *n* (%)

Definition of abbreviations: BMI = body mass index; mMRC, Modified Medical Research Council; CAT, COPD Assessment Test;

* Exacerbations included mild, moderate, and severe exacerbations

**Table 2:** Difference in lung function between participants with and without IOS-BDR in overall participants

	Overall participants (*n* = 466)	Participants without IOS-BDR (*n* = 374)	Participants with IOS-BDR (*n* = 92)	*P*-Value
**Pre-bronchodilator**				
***Spirometry parameters***				
FEV_1_, L	2.09 ± 0.59	2.20 ± 0.56	1.62 ± 0.42	**< 0.001**
FVC, L	3.19 ± 0.72	3.28 ± 0.73	2.86 ± 0.58	**< 0.001**
FEV_1_, %pred	79.6 ± 20.2	83.6 ± 18.8	63.2 ± 17.2	**< 0.001**
FVC, %pred	96.6 ± 16.7	98.8 ± 16.5	87.8 ± 14.2	**< 0.001**
FEV_1_/FVC, %	65.6 ± 12.3	67.5 ± 11.2	57.6 ± 13.2	**< 0.001**
FEF_25 − 75_, % pred	40.2 ± 23.3	44.0 ± 23.4	24.6 ± 15.0	**< 0.001**
FEF_50_, % pred	45.0 ± 26.9	49.5 ± 27.0	26.4 ± 16.7	**< 0.001**
FEF_75_, % pred	31.3 ± 19.6	34.0 ± 20.2	20.1 ± 11.5	**< 0.001**
***IOS parameters***				
R5	0.33 (0.27, 0.39)	0.31 (0.26, 0.36)	0.43 (0.38, 0.49)	**< 0.001**
R20	0.27 (0.23, 0.31)	0.26 (0.23, 0.30)	0.30 (0.26, 0.34)	**< 0.001**
R5-R20	0.05 (0.03, 0.08)	0.04 (0.02, 0.06)	0.14 (0.08, 0.17)	**< 0.001**
X5	-0.12 (-0.16, -0.09)	-0.11 (-0.14, -0.09)	-0.19 (-0.24, -0.15)	**< 0.001**
AX	0.41 (0.22, 0.78)	0.33 (0.19, 0.53)	1.43 (0.85, 1.86)	**< 0.001**
Fres	14.89 (11.12, 18.34)	13.61 (10.60, 16.02)	22.01 (18.69, 24.20)	**< 0.001**
**Post-bronchodilator**				
***Spirometry parameters***				
FEV_1_, L	2.19 ± 0.59	2.29 ± 0.57	1.78 ± 0.45	**< 0.001**
FVC, L	3.26 ± 0.73	3.24 ± 0.74	3.37 ± 0.70	0.121
FEV_1_, %pred	83.6 ± 19.7	87.1 ± 18.6	69.3 ± 17.2	**< 0.001**
FVC, %pred	98.5 ± 16.2	100.1 ± 16.4	91.9 ± 13.7	**< 0.001**
FEV_1_/FVC, %	67.8 ± 12.4	69.5 ± 11.5	60.5 ± 13.3	**< 0.001**
FEF_25 − 75_, % pred	45.8 ± 26.0	49.7 ± 26.4	29.9 ± 16.8	**< 0.001**
FEF_50_, % pred	50.7 ± 28.8	55.3 ± 28.9	32.0 ± 19.4	**< 0.001**
FEF_75_, % pred	36.1 ± 22.9	39.0 ± 23.9	24.4 ± 12.7	**< 0.001**
***IOS parameters***				
R5	0.29 (0.25, 0.35)	0.28 (0.24, 0.33)	0.35 (0.31, 0.40)	**< 0.001**
R20	0.25 (0.22, 0.29)	0.25 (0.21, 0.28)	0.27 (0.24, 0.31)	**< 0.001**
R5-R20	0.04 (0.02, 0.07)	0.03 (0.02, 0.05)	0.08 (0.04, 0.11)	**< 0.001**
X5	-0.11 (-0.14, -0.08)	-0.10 (-0.12, -0.08)	-0.13 (-0.17, -0.10)	**< 0.001**
AX	0.29 (0.18, 0.50)	0.25 (0.16, 0.42)	0.54 (0.34, 0.96)	**< 0.001**
Fres	13.18 (10.24, 15.80)	12.07 (9.66, 14.91)	16.17 (13.99, 20.48)	**< 0.001**
**Absolute change in** ***IOS parameters***				
ΔR5	-0.03 (-0.06, -0.01)	-0.02 (-0.05, 0)	-0.08 (-0.12, -0.06)	**< 0.001**
ΔR20	-0.02 (-0.04, 0)	-0.02 (-0.03, 0)	-0.03 (-0.05, -0.01)	**0.001**
ΔR5-R20	-0.01 (-0.03, 0)	-0.01 (-0.02, 0)	-0.05 (-0.07, -0.03)	**< 0.001**
ΔX5	0.01 (0, 0.03)	0.01 (0, 0.02)	0.05 (0.04, 0.07)	**< 0.001**
ΔAX	-0.09 (-0.29, -0.01)	-0.05 (-0.14, 0)	-0.62 (-0.93, -0.46)	**< 0.001**
ΔFres	-1.13 (-2.96, -0.09)	-0.82 (-2.00, 0.05)	-4.17 (-5.83, -2.19)	**< 0.001**

Data are mean (standard deviation) or *n* (%) or medians (interquartile range)

Definition of abbreviations: IOS, Impulse oscillometry; BDR, bronchodilator response; FEV_1_, forced expiratory volume in one second; FVC, forced vital capacity; R5, resistance at 5 Hz; R20, resistance at 20 Hz; R5-R20, difference from R5 to R20; X5, reactance at 5 Hz; AX, area under the reactance curve; Fres, resonant frequency. Δ: The absolute change was expressed as post-bronchodilator value minus pre-bronchodilator value

### Proportion of IOS-BDR in participants stratified by sex, smoking status, and COPD

**Fig. 2:**
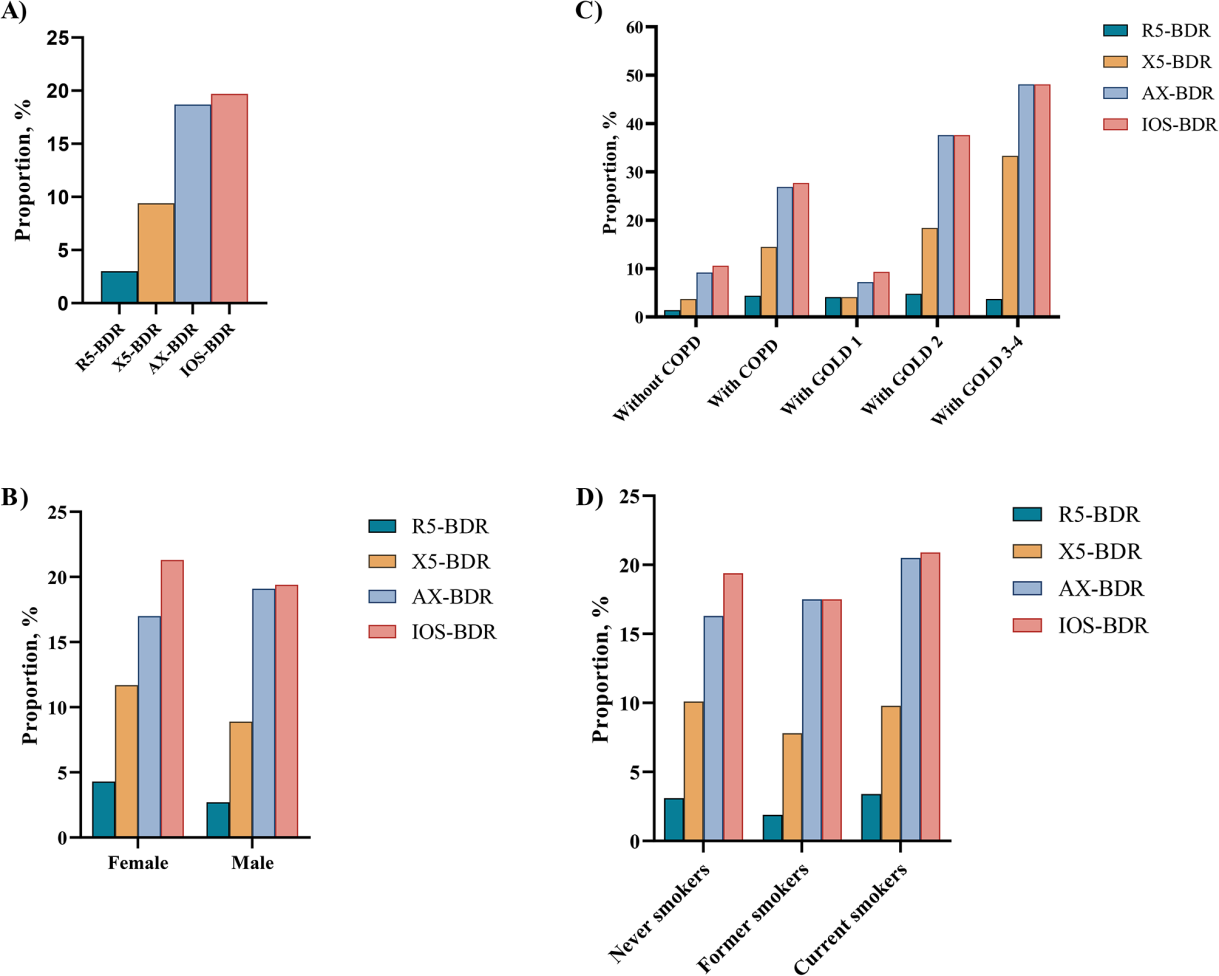
Proportion of IOS-BDR in participants stratified by sex, smoking status, and COPD. (A) in overall participants; (B) in male and female participants; (C) in participants with and without COPD. D)in participants with never smokers, former smokers, current smokers. R5-BDR, bronchodilator response assessed by R5; X5-BDR, bronchodilator response assessed by X5; AX-BDR, bronchodilator response assessed by AX; IOS-BDR, bronchodilator response assessed by one of three parameters (R5, X5, and AX)

Figure 2 depicts the proportion of IOS-BDR in this study. Overall, the proportion of BDR assessed by R5 (R5-BDR), X5 (X5-BDR), AX (AX-BDR), and any of three IOS parameters (IOS-BDR) was 3.0%, 9.4%, 18.7%, and 19.7%, respectively (Fig. 2A). Furthermore, the proportion of AX-BDR was larger than that of R5-BDR and X5-BDR. The participants with COPD had larger proportions of X5-BDR (14.5% vs. 3.7%), AX-BDR (26.9% vs. 9.2%), and IOS-BDR (27.7% vs. 10.6%) than the participants without COPD. However, the proportion of R5-BDR was not significantly different between the participants with and without COPD. In the participants with COPD, the proportions of X5-BDR, AX-BDR, and IOS-BDR increased with COPD severity, where approximately half of the participants with Global Initiative for Chronic Obstructive Lung Disease (GOLD)3–4 had AX-BDR or IOS-BDR. No difference existed in the proportion of IOS-BDR between the participants with GOLD1 and those without COPD. Moreover, the AX-BDR almost included BDR assessed by other indicators (R5-BDR, X5-BDR) in COPD participants with GOLD3–4 (Fig. 2C, Table S1). Additionally, no difference existed in the proportions of R5-BDR, X5-BDR, AX-BDR, and IOS-BDR according to sex and smoking status (Fig. 2B and D).

### Outcomes of participants with and without IOS-BDR

The differences between participants with and without IOS-BDR were investigated in terms of symptom scores and CT imaging changes. After adjusting for age, sex, BMI, pack–years, smoking status, family history of respiratory diseases, occupational exposure, biomass exposure, and history of asthma, multivariable linear regression of the participants overall demonstrated that the participants with IOS-BDR had higher CAT scores, more severe emphysema, and air trapping than those without IOS-BDR (Table 3).

**Table 3:** Univariate and multivariable linear regression models of outcomes in overall participants with and without IOS-BDR.

Outcome *	Group	Unadjusted			Adjusted ^†^		
		Mean difference	95% CI	*P* Value	Mean difference	95% CI	*P* Value
CAT scores (*n* = 465)	Without IOS-BDR	Reference	-		Reference	-	
	With IOS-BDR	1.33	0.56, 2.10	**0.001**	1.20	0.44, 1.97	**0.002**
LAA_− 950_ (*n* = 327)	Without IOS-BDR	Reference	-		Reference	-	
	With IOS-BDR	1.63	0.29, 2.97	**0.017**	2.04	0.81, 3.28	**0.001**
LAA_− 856_ (*n* = 327)	Without IOS-BDR	Reference	-		Reference	-	
	With IOS-BDR	8.28	3.14, 13.43	**0.002**	10.42	6.01, 14.84	**< 0.001**

CAT, COPD Assessment Test; Reference group = Without IOS-BDR.; LAA_− 950_, low-attenuation area of the lung with attenuation values below − 950 Hounsfield units; HU, Hounsfield Unit; LAA_− 856_, low-attenuation area of the lung with attenuation values below − 856 Hounsfield units;

* Linear regression model

† Adjusted for age, sex, BMI, smoking status, pack-years, family history of respiratory diseases, occupation exposure, biomass exposure, history of asthma

### Associations between lung function decline, acute respiratory exacerbations, and IOS-BDR

The associations between lung function decline, acute respiratory exacerbations, and IOS-BDR were observed. In the overall participants, linear mixed-effect model results demonstrated that the participants with IOS-BDR have an increase in decline in post-bronchodilator FVC (mean difference = − 209.1 mL, 95% CI: -329.7 mL, − 88.5 mL, *P* < 0.001) and FEV_1_/FVC (mean difference = − 1.0%, 95% CI: − 1.9%, − 0.2%, *P* = 0.013) than those without IOS-BDR over two visits. After adjusting for age, sex, BMI, pack–years, smoking status, family history of respiratory diseases, occupation exposure, biomass exposure, history of asthma, and post-bronchodilator baseline function (FEV_1_, FVC, FEV_1_/FVC), we found that participants with IOS-BDR have an increase in decline in post-bronchodilator FVC (adjusted mean difference = − 209.3 mL, 95% CI: -339.3 mL, − 79.4 mL, *P* = 0.002), but no difference between participants with and without IOS-BDR in decline in post-bronchodilator FEV_1_/FVC (adjusted mean difference = − 1.0%, 95% CI: -2.0%, 0.03%, *P* = 0.057). Logistic regression model results indicated no differences in any respiratory exacerbations or moderate to severe exacerbations at the 2-year follow-up between the participants with and without IOS-BDR (Table 4).

**Table 4:** Association between acute respiratory exacerbations, decline in lung function and IOS-BDR in overall participants

Outcome	Without IOS-BDR	With IOS-BDR	Unadjusted		Adjusted	
Exacerbations*			**Odds ratio (95% CI)**	*P * **Value**	**Odds ratio (95% CI)** ^†^	*P* **Value**
Any respiratory exacerbations (n=397)	177 (55.5)	46 (59.0)	1.15 (0.70, 1.91)	0.578	0.77 (0.42, 1.41)	0.399
Moderate to severe exacerbations (n=390) ¶	122 (39.0)	39 (50.6)	1.61 (0.97, 2.65)	0.064	1.12 (0.61, 2.05)	0.708
***Annualized lung function*** ^***‡***^			**Mean difference (95% CI)**	*P * **Value**	**Mean difference (95% CI)** ^§^	*P * **Value**
**Post-bronchodilator Spirometry**	N=332	N=80				
Decline in FEV_1,_ mL/y	-38.6 ± 7.6	-56.7 ± 15.3	-18.1 (-49.0, 12.9)	0.251	-12.9 (-131.6, 105.7)	0.831
Decline in FVC, mL/y	12.7 ± 29.7	-198.7 ± 59.7	-209.1 (-329.7, -88.5)	**< 0.001**	-209.3 (-339.3, -79.4)	**0.002**
Decline in FEV_1_/FVC, %/y	-0.5 ± 0.2	-1.6 ± 0.4	-1.0 (-1.9, -0.2)	**0.013**	-1.0 (-2.0, 0.03)	0.057

IOS, Impulse oscillometry; BDR, bronchodilator response; FEV_1_, forced expiratory volume in one second; FVC, forced vital capacity; COPD, Chronic Obstructive Pulmonary Disease

* Logistic regression model; exacerbations at 2-year follow-up as binary variable 0 vs. ≥ 1

† Adjusted for age, sex, BMI, smoking status, pack-years, family history of respiratory diseases, occupation exposure, biomass exposure, history of asthma, exacerbations in previous year and baseline pre-bronchodilator FEV_1_.

‡ Linear mixed-effects model

§ Adjusted for age, sex, BMI, smoking status, pack-years, family history of respiratory diseases, occupation exposure, biomass exposure, history of asthma and baseline lung function (FEV_1_, FVC, and FEV_1_/FVC).

¶ 7 pariticipants without Moderate to severe exacerbations data

### Subgroup analyses results

The associations between symptom scores, emphysema, air trapping, lung function decline, exacerbations, and IOS-BDR were examined with stratified analyses stratified by sex, smoking status, and COPD. The participants with IOS-BDR had higher symptom scores, more severe emphysema, and air trapping than those without IOS-BDR both male and ever-smoker participants (Table S4, Table S6). The participants with IOS-BDR had more severe emphysema, air trapping than those without IOS-BDR both participants with COPD and female participants (Table S2, Table S5). No difference existed between the participants with and without IOS-BDR in terms of symptom scores, emphysema, air trapping among never-smoker and the participants without COPD (Table S3 and Table S7). The participants with IOS-BDR had an increase in decline in post-bronchodilator FVC than those without IOS-BDR among male participants, never and ever-smoker participants, participants with and without COPD (Table S8-10, S12-13). However, no difference existed between the participants with and without IOS-BDR in terms of decline in post-bronchodilator FVC, and acute respiratory exacerbations among female participants (Table S11).

## Discussion

This study describes the clinical characterization of IOS-BDR in participants from a general population. The participants with IOS-BDR exhibited more respiratory symptoms, emphysema, and air trapping than the participants without IOS-BDR. The longitudinal analysis demonstrated that IOS-BDR was associated with decline in lung function but unrelated to the risk of acute exacerbations.

In this study, the proportions of R5-BDR, X5-BDR, AX-BDR, and IOS-BDR were 3.0%, 9.4%, 18.7%, and 19.7%, respectively, in the overall participants. These results suggested that AX-defined BDR might better detecte more small airway responsiveness than R5-BDR and X5-BDR. Subsequently, the proportion of BDR assessed by IOS parameters was explored in different participants. No difference existed in the proportions of R5-BDR, X5-BDR, AX-BDR, and IOS-BDR when the participants were stratified by sex and smoking status. The proportion of IOS-BDR was 10.6% in the participants without COPD and was higher (27.7%) in the participants with COPD. BDR assessment using X5 and AX yielded similar results. However, the proportion of R5-BDR was not statistically significantly different between the participants with and without COPD. This results suggested that respiratory system reactance (Xrs) may be more sensitive than respiratory system resistance (Rrs) for detecting small airway responsiveness in COPD patients [22]. The reason may be that Xrs reflected stiffnesses of the lung and chest wall tissues, and may sensitivly detecte airway closure and severe narrowing in COPD [36].

The proportion of IOS-BDR gradually increased with COPD severity, where nearly half of the COPD participants with GOLD3–4 had IOS-BDR. However, the proportion of IOS-BDR between the COPD participants with GOLD 1 and participants without COPD was not statistically significantly different. The results revealed less IOS-BDR in early-stage COPD, especially in participants with mild COPD, but the IOS-BDR increased with disease progressions. Thus, IOS-BDR was associated with COPD severity.

In patients with advanced COPD, airway remodeling and emphysema, accompanied by loss of alveolar attachment, lead to early expiratory collapse of the small airway, followed by air trapping and dynamic hyperinflation. Stephen et al. reported that BDR assessed by forced oscillation was associated with hyperinflation and gas trapping in COPD [40]. An increased proportion of IOS-BDR closely reflects the progression of emphysema and small airway disease. The results of the present study confirmed this viewpoint, where the participants with IOS-BDR exhibited more severe emphysema and air trapping by high-resolution CT compared to those without IOS-BDR.

Alobaidi et al. reported that small airway BDR was defined based on a change in maximum mid-expiratory flow (MMEF) ≥ 30% and change ≥ 12% and absolute change ≥ 200 mL in the FEV_1_. Alobaidi et al. reported that MMEF detected a certain proportion of BDR in participants without BDR assessed by FEV_1_, suggesting that small airway BDR might benefit from the different treatable characteristics subtype [41].

To our knowledge, this is the first prospective study to reveal an association between respiratory symptoms, acute respiratory exacerbations, and decline in lung function and IOS-BDR. At baseline, the participants with IOS-BDR had more cough, wheeze, history of asthma, and medication use than those without IOS-BDR. These findings suggested that IOS-BDR was potentially associated with asthma. However, after adjusting for a history of asthma, the participants with IOS-BDR had higher mMRC and CAT scores than those without IOS-BDR. It is believed that IOS-BDR might reflect dynamic hyperinflation and premature airway closure, which can result in dyspnea. Accordingly, IOS-BDR might reflect the signs of early or subclinical COPD.

To confirm this hypothesis, the difference between participants with and without IOS-BDR in terms of lung function decline and acute respiratory exacerbations was analyzed. The participants with IOS-BDR had a rapid decline in FVC than those without IOS-BDR in the participants with COPD. This result indicated that IOS-BDR might reflect a special COPD subtype. Numerous studies demonstrated that patients with spirometric BDR experienced a rapid decline in lung function than patients without spirometric BDR. However, after adjusting for baseline FEV_1_, the spirometric BDR demonstrated no association with lung function decline [42, 43]. Nevertheless, this study determined that, after adjusting for baseline lung function, the participants with IOS-BDR persistently exhibited a rapid decline in lung function compared with those without IOS-BDR. This result suggested that IOS-BDR might reflect different physiological characteristics compared with spirometric BDR.

Previous research demonstrated that BDR might indicate inflammation and be associated with eosinophil changes and increased exhaled nitric oxide [44, 45]. Patients with IOS-BDR might respond well after inhaling corticosteroids. Therefore, early treatment with inhaled corticosteroids (ICS) in COPD patients with IOS-BDR might effectively impede the decline in lung function.

Among the participants without COPD, 10.6% paticipants had IOS-BDR. Here, Xrs exhibited more significant changes compared to Rrs after the administration of 400 µg salbutamol. This finding contradicted previous research that reported a decrease in Rrs but non-significant changes in Xrs in healthy participants after inhaling bronchodilators [19, 46] A possible explanation is that an increase in the proportion of IOS-BDR might be associated with respiratory symptoms. While Oostveen et al. enrolled asymptomatic healthy participants without cardiopulmonary diseases, the present study enrolled some symptomatic participants, and the baseline results demonstrated that participants with IOS-BDR had more cough and wheezing symptoms, and higher CAT scores and mMRC scores than the participants without IOS-BDR. Jetmalani et al. also demonstrated a higher proportion of IOS-BDR in smoking individuals with respiratory symptoms than in asymptomatic smoking individuals, and the proportion of BDR assessed by Rrs and Xrs was similar in asymptomatic healthy participants (∼5.0%) [22] .

Previous study has identified differences in IOS parameters but spirometry indicators showed no differences before and after bronchodilator inhalation in health individuals. This result suggested that, in the early stages of COPD, IOS may be more sensitive in detecting airway responsiveness compared to traditional spirometry [23]. Our findings showed that in the participants without COPD, IOS-BDR was associated with lung function decline after adjusting for covariates. This result implied that individuals with IOS-BDR may be higher risk participants in pre-COPD. Early intervention may potentially slow down the decline in lung function and prevent progression to COPD. Similar to the spirometric BDR outcome in many studies, the present study detected no association between IOS-BDR and the risk of acute respiratory events/exacerbations in patients with COPD [6, 47]. Further studies are warranted to identify the underlying mechanisms of IOS-BDR in patients without COPD.

This study had some limitations. First, IOS-BDR was defined as the absolute change in IOS parameters in our study. However, the absolute value strongly depended on the baseline value, increasing the proportion of IOS-BDR. The relative changes or Z-score changes in IOS parameters were recommended to greatly reflect BDR, but almost no participants with IOS-BDR defined based on the relative IOS parameter changes were detected in this study (not shown). In the present study, it is believed that many participants with mild to moderate COPD with low airway resistance after bronchodilator administration might not respond well. Accordingly, the recommended threshold of relative changes might be unsuitable for participants with COPD, and new thresholds should be explored for assessing IOS-BDR. Second, given the lack of information on ICS/long-acting β2-agonist (LABA) treatment, whether ICS/LABA use would affect the prognosis remained unclear. Thirdly, due to the greater variability of IOS compared with spirometric parameters [48, 49], previous studies have reported that there was individual variability and day instability in spirometric BDR [3, 50], however, the individual variability of IOS-BDR and whether IOS-BDR would identify a useful phenotype remained unclear. In addition, single IOS measurements was used in this study, different devices will be included to analyze the robustness of the results in future. Finally,We are sorry that design of the ECOPD cohort did not include the information related to Corona Virus Disease 2019 (COVID-19) infection, the reasons were as follow: (1) The contents of COVID-19 were not collected in design of the ECOPD cohort study. (2) At the end of 2022, there is no way to obtain accurate results due to none conditions for nasopharyngeal swab in some places. Although the contents of COVID-19 infection were not collected, we believe that COVID-2019 infection has little impact on the results of this study. at the end of 2022, it reported spread of the SARS-CoV2 Omicron variant in a very large population of very low pre-existing immunity, among hospitalized patients with Omicron infection olny had mild disease [51, 52]. In addition, participants were required to perform lung function tests only when no acute exacerbation or acute upper respiratory tract infection occurred one month before the follow-up to ensure the accuracy of lung function.

## Conclusion

IOS-BDR was prevalent in the participants with COPD, especially those with GOLD3–4. Participants with IOS-BDR had more respiratory symptoms, radiographic structural changes, and a rapid decline in lung function than those without IOS-BDR, suggesting that IOS-BDR might benefit from the different treatable characteristic subtypes.

### Electronic supplementary material

Below is the link to the electronic supplementary material.

Supplementary Material 1

## Data Availability

No datasets were generated or analysed during the current study.

## Abbreviations

IOS: Impulse oscillometry
SAD: Small airway dysfunction
FEV_1_: Forced expiratory volume in one second
FVC: Forced vital capacity
BDR: Bronchodilator response
R5: Resistance at 5 Hz
R5: R20-difference from R5 to R20
X5: Reactance at 5 Hz
AX: Area under the reactance curve
Fres: Resonant frequency
