# Supplementary material for: Clinical characterization and outcomes of impulse oscillometry-defined bronchodilator response: an ECOPD cohort-based study
Source: Respir Res. 2024 Mar 30;25:149. doi: 10.1186/s12931-024-02765-7 (PMC10981824; doi:10.1186/s12931-024-02765-7)
Supplement: Supplementary file 1 — Supplementary Material 1 [file 12931_2024_2765_MOESM1_ESM.docx]

**Table S1**. Proportion of IOS-BDR participants with and without COPD.

| Small airway BDR | All subjects  (n=466) | Without COPD  (n=217) | COPD  (n=249) | GOLD 1  (n=97) | GOLD 2  (n=125) | GOLD 3-4  (n=27) | P-value |
| --- | --- | --- | --- | --- | --- | --- | --- |
| R5-BDR, n (%) | 14 (3.0) | 3 (1.4) | 11 (4.4) | 4 (4.1) | 6 (4.8) | 1 (3.7) | 0.283 |
| X5-BDR, n (%) | 44 (9.4) | 8 (3.7) | 36 (14.5) ^a^ | 4 (4.1) | 23 (18.4) ^a, b^ | 9 (33.3) ^a, b^ | < 0.001 |
| AX-BDR, n (%) | 87 (18.7) | 20 (9.2) | 67 (26.9) ^a^ | 7 (7.2) | 47 (37.6) ^a, b^ | 13 (48.1) ^a, b^ | < 0.001 |
| IOS-BDR, n (%) | 92 (19.7) | 23 (10.6) | 69 (27.7) ^a^ | 9 (9.3) | 47 (37.6) ^a, b^ | 13 (48.1) ^a, b^ | < 0.001 |

Data are shown as n (%), Definition of abbreviations: R5-BDR, bronchodilator response assessed by R5; X5-BDR, bronchodilator response assessed by X5; AX-BDR, bronchodilator response assessed by AX; IOS-BDR, bronchodilator response assessed by one of three parameters (R5, X5, AX).

a: compare with subjects without COPD

b: compare with subjects with GOLD 1

**Table** **S2**. Univariate and multivariable linear models of outcomes in COPD participants with and without IOS-BDR.

|  |  | **Unadjusted** | |  | **Adjusted †** | |
| --- | --- | --- | --- | --- | --- | --- |
| **Outcome *** | **Group** | **Mean difference (95% CI)** | **P Value** |  | **Mean difference (95% CI)** | **P Value** |
| CAT scores (n=247) | Without IOS-BDR | Reference |  |  | Reference |  |
|  | With IOS-BDR | 0.92 (-0.12, 1.97) | 0.083 |  | 1.05 (-0.01, 2.11) | 0.053 |
|  |  |  |  |  |  |  |
| LAA_-950_ (n=147) | Without IOS-BDR | Reference |  |  | Reference |  |
|  | With IOS-BDR | 0.62 (-1.61, 2.84) | 0.584 |  | 2.31 (0.11, 4.51) | **0.040** |
|  |  |  |  |  |  |  |
| LAA_-856_ (n=147) | Without IOS-BDR | Reference |  |  | Reference |  |
|  | With IOS-BDR | 4.81 (-2.45, 12.06) | 0.193 |  | 11.74 (4.89, 18.60) | **0.001** |

CAT, COPD Assessment Test; Reference group = Without IOS-BDR.; LAA_−950_, low-attenuation area of the lung with attenuation values below −950 Hounsfield units; HU, Hounsfield Unit; LAA_−856_, low-attenuation area of the lung with attenuation values below −856 Hounsfield units;

* Linear regression model.

† Adjusted for age, sex, BMI, smoking status, pack-years, family history of respiratory diseases, occupation exposure, biomass exposure, history of asthma.

**Table** **S3**. Univariate and multivariable linear models of outcomes in participants without COPD with and without IOS-BDR.

|  |  | **Unadjusted** | |  | **Adjusted ^†^** | |
| --- | --- | --- | --- | --- | --- | --- |
| **Outcome *** | **Group** | **Mean difference (95% CI)** | **P Value** |  | **Mean difference (95% CI)** | **P Value** |
| CAT scores (n=216) | Without IOS-BDR | Reference |  |  | Reference |  |
|  | With IOS-BDR | 1.13 (-0.06, 2.33) | 0.064 |  | 1.17 (-0.09, 2.42) | 0.067 |
|  |  |  |  |  |  |  |
| LAA_-950_ (n=178) | Without IOS-BDR | Reference |  |  | Reference |  |
|  | With IOS-BDR | 0.26 (-0.43, 0.95) | 0.460 |  | 0.47 (-0.16, 1,11) | 0.144 |
|  |  |  |  |  |  |  |
| LAA_-856_ (n=178) | Without IOS-BDR | Reference |  |  | Reference |  |
|  | With IOS-BDR | -0.93 (-5.72, 3.87) | 0.704 |  | 0.43 (-3.92, 4.79) | 0.844 |

CAT, COPD Assessment Test; Reference group = Without IOS-BDR.; LAA_−950_, low-attenuation area of the lung with attenuation values below −950 Hounsfield units; HU, Hounsfield Unit; LAA_−856_, low-attenuation area of the lung with attenuation values below −856 Hounsfield units;

* Linear regression model.

† Adjusted for age, sex, BMI, smoking status, pack-years, family history of respiratory diseases, occupation exposure, biomass exposure, history of asthma.

**Table S4**. Univariate and multivariable linear models of outcomes in male participants with and without IOS-BDR.

|  |  | **Unadjusted** | |  | **Adjusted ^†^** | |
| --- | --- | --- | --- | --- | --- | --- |
| **Outcome *** | **Group** | **Mean difference (95% CI)** | **P Value** |  | **Mean difference (95% CI)** | **P Value** |
| CAT scores (n=370) | Without IOS-BDR | Reference |  |  | Reference |  |
|  | With IOS-BDR | 1.42 (0.53, 2.32) | **0.002** |  | 1.27 (0.39, 2.15) | **0.005** |
|  |  |  |  |  |  |  |
| LAA-950 (n=256) | Without IOS-BDR | Reference |  |  | Reference |  |
|  | With IOS-BDR | 2.29 (0.61, 3.97) | **0.008** |  | 2.93 (1.34, 4.52) | **< 0.001** |
|  |  |  |  |  |  |  |
| LAA_-856_ (n=256) | Without IOS-BDR | Reference |  |  | Reference |  |
|  | With IOS-BDR | 10.50 (4.22, 16.77) | **0.001** |  | 13.39 (7.81, 18.96) | **< 0.001** |

CAT, COPD Assessment Test; Reference group = Without IOS-BDR.; LAA_−950_, low-attenuation area of the lung with attenuation values below −950 Hounsfield units; HU, Hounsfield Unit; LAA_−856_, low-attenuation area of the lung with attenuation values below −856 Hounsfield units;

* Linear regression model.

† Adjusted for age, sex, BMI, smoking status, pack-years, family history of respiratory diseases, occupation exposure, biomass exposure, history of asthma.

**Table** S**5**. Univariate and multivariable linear models of outcomes in female participants with and without IOS-BDR.

|  |  | **Unadjusted** | |  | **Adjusted ^†^** | |
| --- | --- | --- | --- | --- | --- | --- |
| **Outcome *** | **Group** | **Mean difference (95% CI)** | **P Value** |  | **Mean difference (95% CI)** | **P Value** |
| CAT scores (n=93) | Without IOS-BDR | Reference |  |  | Reference |  |
|  | With IOS-BDR | 1.07 (-0.33, 2.47) | 0.132 |  | 0.96 (-0.54, 2.47) | 0.207 |
|  |  |  |  |  |  |  |
| LAA_-950_ (n=69) | Without IOS-BDR | Reference |  |  | Reference |  |
|  | With IOS-BDR | 0.31 (0.10, 0.52) | **0.004** |  | 0.41 (0.20, 0.61) | **< 0.001** |
|  |  |  |  |  |  |  |
| LAA_-856_ (n=69) | Without IOS-BDR | Reference |  |  | Reference |  |
|  | With IOS-BDR | 4.53 (1.08, 7.97) | **0.011** |  | 5.35 (2.17, 8.52) | **0.001** |

CAT, COPD Assessment Test; Reference group = Without IOS-BDR.; LAA_−950_, low-attenuation area of the lung with attenuation values below −950 Hounsfield units; HU, Hounsfield Unit; LAA_−856_, low-attenuation area of the lung with attenuation values below −856 Hounsfield units;

* Linear regression model.

† Adjusted for age, sex, BMI, smoking status, pack-years, family history of respiratory diseases, occupation exposure, biomass exposure, history of asthma.

**Table** **S6**. Univariate and multivariable linear models of outcomes in ever-smoker participants with and without IOS-BDR.

|  |  | **Unadjusted** | |  | **Adjusted ^†^** | |
| --- | --- | --- | --- | --- | --- | --- |
| **Outcome *** | **Group** | **Mean difference (95% CI)** | **P Value** |  | **Mean difference (95% CI)** | **P Value** |
| CAT scores (n=335) | Without IOS-BDR | Reference |  |  | Reference |  |
|  | With IOS-BDR | 1.55 (0.60, 2.50) | **0.001** |  | 1.46 (0.52, 2.40) | **0.002** |
|  |  |  |  |  |  |  |
| LAA_-950_ (n=228) | Without IOS-BDR | Reference |  |  | Reference |  |
|  | With IOS-BDR | 2.43 (0.61, 4.25) | **0.009** |  | 3.21 (1.50, 4.92) | **< 0.001** |
|  |  |  |  |  |  |  |
| LAA_-856_ (n=228) | Without IOS-BDR | Reference |  |  | Reference |  |
|  | With IOS-BDR | 11.24 (4.73, 17.74) | **0.001** |  | 14.65 (8.91, 20.40) | **< 0.001** |

CAT, COPD Assessment Test; Reference group = Without IOS-BDR.; LAA_−950_, low-attenuation area of the lung with attenuation values below −950 Hounsfield units; HU, Hounsfield Unit; LAA_−856_, low-attenuation area of the lung with attenuation values below −856 Hounsfield units;

* Linear regression model.

† Adjusted for age, sex, BMI, smoking status, pack-years, family history of respiratory diseases, occupation exposure, biomass exposure, history of asthma.

**Table** **S7**. Univariate and multivariable linear models of outcomes in never-smoker participants with and without IOS-BDR.

|  |  | **Unadjusted** | |  | **Adjusted ^†^** | |
| --- | --- | --- | --- | --- | --- | --- |
| **Outcome *** | **Group** | **Mean difference (95% CI)** | **P Value** |  | **Mean difference (95% CI)** | **P Value** |
| CAT scores (n=128) | Without IOS-BDR | Reference |  |  | Reference |  |
|  | With IOS-BDR | 0.70 (-0.46, 1.86) | 0.237 |  | 0.53 (-0.70, 1.75) | 0.396 |
|  |  |  |  |  |  |  |
| LAA_-950_ (n=97) | Without IOS-BDR | Reference |  |  | Reference |  |
|  | With IOS-BDR | 0.10 (-0.84, 1.05) | 0.833 |  | 0.35 (-0.49, 1.20) | 0.409 |
|  |  |  |  |  |  |  |
| LAA_-856_ (n=97) | Without IOS-BDR | Reference |  |  | Reference |  |
|  | With IOS-BDR | 2.71 (-3.78, 9.21) | 0.409 |  | 4.39 (-1.46, 10.25) | 0.140 |

CAT, COPD Assessment Test; Reference group = Without IOS-BDR.; LAA_−950_, low-attenuation area of the lung with attenuation values below −950 Hounsfield units; HU, Hounsfield Unit; LAA_−856_, low-attenuation area of the lung with attenuation values below −856 Hounsfield units;

* Linear regression model.

† Adjusted for age, sex, BMI, smoking status, pack-years, family history of respiratory diseases, occupation exposure, biomass exposure, history of asthma.

**Table** **S8.** Association between exacerbations, decline in spirometry-defined lung function and IOS-BDR in participants with COPD.

| **Outcome** | **Without IOS-BDR** | **With IOS-BDR** | **Unadjusted** |  | **Adjusted** |  |
| --- | --- | --- | --- | --- | --- | --- |
| ***Exacerbations**** |  |  | **Odds ratio (95% CI)** | **P Value** | **Odds ratio (95% CI)** ^†^ | **P Value** |
| Any respiratory exacerbations (n=209) | 87 (58.8) | 37 (60.7) | 1.08 (0.59, 1.99) | 0.802 | 0.86 (0.41, 1.81) | 0.699 |
| Moderate to severe exacerbations (n=205)^¶^ | 61 (42.1) | 33 (55.0) | 1.68 (0.92, 3.09) | 0.092 | 1.27 (0.60, 2.68) | 0.526 |
| ***Annualized lung function***^‡^ |  |  | **Mean difference (95% CI)** | **P Value** | **Mean difference (95% CI)**^§^ | **P Value** |
| **Post-bronchodilator Spirometry** | N=158 | N=63 |  |  |  |  |
| Decline in FEV_1,_ mL/y | -41.8 + 8.9 | -51.9 + 13.8 | -8.8 (-44.5, 26.9) | 0.627 | -1.1 (-142.4, 140.1) | 0.988 |
| Decline in FVC, mL/y | 57.0 + 40.8 | -111.6 + 64.5 | -165.6 (-313.9, -17.3) | **0.029** | -164.5 (-321.4, -7.5) | **0.040** |
| Decline in FEV_1_/FVC, %/y | -0.3 + 0.2 | -1.0 + 0.4 | -0.7 (-1.5, 0.2) | 0.113 | -0.6 (-1.8, 0.6) | 0.310 |

IOS, Impulse oscillometry; BDR, bronchodilator response; FEV_1_, forced expiratory volume in one second; FVC, forced vital capacity; COPD, Chronic Obstructive Pulmonary Disease.

* Logistic regression model; exacerbations at 2-year follow-up as binary variable 0 vs ≥ 1.

†Adjusted for age, sex, BMI, smoking status, pack-years, family history of respiratory diseases, occupation exposure, biomass exposure, history of asthma, exacerbations in previous year and baseline pre-bronchodilator FEV_1_.

‡linear mixed-effects model.

§ Adjusted for age, sex, BMI, smoking status, pack-years, family history of respiratory diseases, occupation exposure, biomass exposure, history of asthma and baseline lung function (FEV_1_, FVC, and FEV_1_/FVC).

¶ 4 pariticipants without Moderate to severe exacerbations data

**Table S9.** Association between exacerbations, decline in spirometry-defined lung function and IOS-BDR in participants without COPD.

| **Outcome** | Without IOS-BDR | With IOS-BDR | **Unadjusted** |  | **Adjusted** |  |
| --- | --- | --- | --- | --- | --- | --- |
| ***Exacerbations**** |  |  | **Odds ratio (95% CI)** | **P Value** | **Odds ratio (95% CI)** ^†^ | **P Value** |
| Any respiratory exacerbations (n=188) | 90 (52.6) | 9 (52.9) | 1.01 (0.37, 2.75) | 0.981 | 0.56 (0.17, 1.85) | 0.344 |
| Moderate to severe exacerbations (n=185)^¶^ | 61 (36.3) | 6 (35.3) | 0.96 (0.34, 2.72) | 0.934 | 0.64 (0.18, 2.22) | 0.480 |
| ***Annualized lung function***^‡^ |  |  | **Mean difference (95% CI)** | **P Value** | **Mean difference (95% CI)**^§^ | **P Value** |
| **Post-bronchodilator Spirometry** | N=174 | N=17 |  |  |  |  |
| Decline in FEV_1,_ mL/y | -34.6 + 10.3 | -76.2 + 31.8 | -41.6 (-107.6, 24.4) | 0.215 | -44.0 (-164.9, 76.9) | 0.475 |
| Decline in FVC, mL/y | -27.5 + 36.0 | -514.2 + 110.1 | -486.7 (-715.0, -258.4) | **< 0.001** | -478.2 (-704.7, -251.8) | **< 0.001** |
| Decline in FEV_1_/FVC, %/y | -0.7 + 0.3 | -3.2 + 0.9 | -2.5 (-4.4, -0.7) | **0.008** | -2.5 (-4.6, -0.5) | **0.016** |

IOS, Impulse oscillometry; BDR, bronchodilator response; FEV_1_, forced expiratory volume in one second; FVC, forced vital capacity; COPD, Chronic Obstructive Pulmonary Disease.

* Logistic regression model; exacerbations at 2-year follow-up as binary variable 0 vs ≥ 1.

† Adjusted for age, sex, BMI, smoking status, pack-years, family history of respiratory diseases, occupation exposure, biomass exposure, history of asthma, exacerbations in previous year and baseline pre-bronchodilator FEV_1_.

‡linear mixed-effects model.

§ Adjusted for age, sex, BMI, smoking status, pack-years, family history of respiratory diseases, occupation exposure, biomass exposure, history of asthma and baseline lung function (FEV_1_, FVC, and FEV_1_/FVC).

¶ 3 pariticipants without Moderate to severe exacerbations data

**Table S10.** Association between exacerbations, decline in spirometry-defined lung function and IOS-BDR in male participants.

| **Outcome** | Without IOS-BDR | With IOS-BDR | **Unadjusted** |  | **Adjusted** |  |
| --- | --- | --- | --- | --- | --- | --- |
| ***Exacerbations**** |  |  | **Odds ratio (95% CI)** | **P Value** | **Odds ratio (95% CI)** ^†^ | **P Value** |
| Any respiratory exacerbations (n=317) | 137 (53.3) | 33 (55.0) | 1.07 (0.61, 1.88) | 0.813 | 0.80 (0.40, 1.58) | 0.516 |
| Moderate to severe exacerbations (n=312)^¶^ | 92 (36.4) | 29 (49.2) | 1.69 (0.96, 3.00) | 0.071 | 1.32 (0.66, 2.64) | 0.439 |
| ***Annualized lung function***^‡^ |  |  | **Mean difference (95% CI)** | **P Value** | **Mean difference (95% CI)**^§^ | **P Value** |
| **Post-bronchodilator Spirometry** | N= 267 | N= 62 |  |  |  |  |
| Decline in FEV_1,_ mL/y | -42.2 + 8.3 | -64.2 + 17.2 | -22.0 (-59.7, 15.6) | 0.250 | -16.1 (-150.5, 118.3) | 0.814 |
| Decline in FVC, mL/y | 85.5 + 29.3 | -91.3 + 60.2 | -176.8 (-308.5, -45.1) | **0.009** | -180.7 (-323.6, -37.9) | **0.013** |
| Decline in FEV_1_/FVC, %/y | -0.6 + 0.2 | -1.8 + 0.4 | -1.1 (-2.1, -0.2) | **0.023** | -1.0 (-2.1, 0.2) | 0.100 |

IOS, Impulse oscillometry; BDR, bronchodilator response; FEV_1_, forced expiratory volume in one second; FVC, forced vital capacity; COPD, Chronic Obstructive Pulmonary Disease.

* Logistic regression model; exacerbations at 2-year follow-up as binary variable 0 vs ≥ 1.

† Adjusted for age, sex, BMI, smoking status, pack-years, family history of respiratory diseases, occupation exposure, biomass exposure, history of asthma, exacerbations in previous year and baseline pre-bronchodilator FEV_1_.

‡linear mixed-effects model.

§ Adjusted for age, sex, BMI, smoking status, pack-years, family history of respiratory diseases, occupation exposure, biomass exposure, history of asthma and baseline lung function (FEV_1_, FVC, and FEV_1_/FVC).

¶ 5 pariticipants without Moderate to severe exacerbations data

**Table S11.** Association between exacerbations, decline in spirometry-defined lung function and IOS-BDR in female participants.

| **Outcome** | **Without IOS-BDR** | **With IOS-BDR** | **Unadjusted** |  | **Adjusted** |  |
| --- | --- | --- | --- | --- | --- | --- |
| ***Exacerbations**** |  |  | **Odds ratio (95% CI)** | **P Value** | **Odds ratio (95% CI)** ^†^ | **P Value** |
| Any respiratory exacerbations (n=80) | 40 (64.5) | 13 (72.2) | 1.43 (0.45, 4.54) | 0.544 | 0.74 (0.18, 2.97) | 0.667 |
| Moderate to severe exacerbations (n=78)^¶^ | 30 (50.0) | 10 (55.6) | 1.25 (0.43, 3.60) | 0.679 | 0.69 (0.18, 2.72) | 0.595 |
| ***Annualized lung function***^‡^ |  |  | **Mean difference (95% CI)** | **P Value** | **Mean difference (95% CI)**^§^ | **P Value** |
| **Post-bronchodilator Spirometry** | N= 65 | N= 18 |  |  |  |  |
| Decline in FEV_1,_ mL/y | -23.3 + 10.5 | -31.8 + 18.7 | -8.5 (-51.0, 33.9) | 0.692 | -5.7 (-73.7, 62.3) | 0.869 |
| Decline in FVC, mL/y | -302.1 + 54.7 | -559.5 + 101.2 | -257.4 (-486.4, -28.5) | **0.028** | -240.3 (-481.1, 0.6) | 0.051 |
| Decline in FEV_1_/FVC, %/y | 0.1 + 0.3 | -1.0 + 0.6 | -1.0 (-2.4, 0.4) | 0.152 | -1.1 (-3.0, 0.8) | 0.263 |

IOS, Impulse oscillometry; BDR, bronchodilator response; FEV_1_, forced expiratory volume in one second; FVC, forced vital capacity; COPD, Chronic Obstructive Pulmonary Disease.

* Logistic regression model; exacerbations at 2-year follow-up as binary variable 0 vs ≥ 1.

† Adjusted for age, sex, BMI, smoking status, pack-years, family history of respiratory diseases, occupation exposure, biomass exposure, history of asthma, exacerbations in previous year and baseline pre-bronchodilator FEV_1_.

‡linear mixed-effects model.

§ Adjusted for age, sex, BMI, smoking status, pack-years, family history of respiratory diseases, occupation exposure, biomass exposure, history of asthma and baseline lung function (FEV1, FVC, and FEV_1_/FVC).

¶ 2 pariticipants without Moderate to severe exacerbations data

**Table S12.** Association between exacerbations, decline in spirometry-defined lung function and IOS-BDR in ever-smoker participants.

| **Outcome** | **Without IOS-BDR** | **With IOS-BDR** | **Unadjusted** |  | **Adjusted** |  |
| --- | --- | --- | --- | --- | --- | --- |
| ***Exacerbations**** |  |  | **Odds ratio (95% CI)** | **P Value** | **Odds ratio (95% CI)** ^†^ | **P Value** |
| Any respiratory exacerbations (n=288) | 121 (52.4) | 33 (57.9) | 1.25 (0.70, 2.25) | 0.455 | 0.99 (0.49, 2.00) | 0.971 |
| Moderate to severe exacerbations (n=283)^¶^ | 81 (35.7) | 29 (51.8) | 1.94 (1.07, 3.49) | **0.028** | 1.67 (0.81, 3.44) | 0.161 |
| ***Annualized lung function***^‡^ |  |  | **Mean difference (95% CI)** | **P Value** | **Mean difference (95% CI)**^§^ | **P Value** |
| **Post-bronchodilator Spirometry** | N=240 | N=58 |  |  |  |  |
| Decline in FEV_1,_ mL/y | -44.0 + 8.9 | -67.3 + 18.0 | -23.3 (-63.0, 16.4) | 0.249 | -17.2 (-156.7, 122.3) | 0.809 |
| Decline in FVC, mL/y | 67.4 + 31.4 | -86.7 + 63.2 | -154.0 (-292.9, -15.1) | **0.030** | -161.9 (-312.4, -11.5) | **0.035** |
| Decline in FEV_1_/FVC, %/y | -0.7 + 0.2 | -1.9 + 0.5 | -1.2 (-2.2, -0.2) | **0.023** | -1.0 (-2.2, 0.2) | 0.108 |

IOS, Impulse oscillometry; BDR, bronchodilator response; FEV_1_, forced expiratory volume in one second; FVC, forced vital capacity; COPD, Chronic Obstructive Pulmonary Disease.

* Logistic regression model; exacerbations at 2-year follow-up as binary variable 0 vs ≥ 1.

† Adjusted for age, sex, BMI, smoking status, pack-years, family history of respiratory diseases, occupation exposure, biomass exposure, history of asthma, exacerbations in previous year and baseline pre-bronchodilator FEV_1_.

‡linear mixed-effects model.

§ Adjusted for age, sex, BMI, smoking status, pack-years, family history of respiratory diseases, occupation exposure, biomass exposure, history of asthma and baseline lung function (FEV_1_, FVC, and FEV_1_/FVC).

¶ 5 pariticipants without Moderate to severe exacerbations data

**Table S13.** Association between exacerbations, decline in spirometry-defined lung function and IOS-BDR in never-smoker participants.

| **Outcome** | **Without IOS-BDR** | **With IOS-BDR** | **Unadjusted** |  | **Adjusted** |  |
| --- | --- | --- | --- | --- | --- | --- |
| ***Exacerbations**** |  |  | **Odds ratio (95% CI)** | **P Value** | **Odds ratio (95% CI)** ^†^ | **P Value** |
| Any respiratory exacerbations (n=109) | 56 (63.6) | 13 (61.9) | 0.93 (0.35, 2.48) | 0.882 | 0.39 (0.12, 1.32) | 0.130 |
| Moderate to severe exacerbations (n=107)^¶^ | 41 (47.7) | 10 (47.6) | 1.00 (0.38, 2.59) | 0.996 | 0.40 (0.11, 1.39) | 0.148 |
| ***Annualized lung function***^‡^ |  |  | **Mean difference (95% CI)** | **P Value** | **Mean difference (95% CI)**^§^ | **P Value** |
| **Post-bronchodilator Spirometry** | N= 92 | N= 22 |  |  |  |  |
| Decline in FEV_1,_ ml/y | -24.3 + 9.2 | -28.8 + 18.1 | -4.5 (-44.9, 35.9) | 0.825 | -2.4 (-112.4, 107.5) | 0.966 |
| Decline in FVC, ml/y | -142.0 + 51.6 | -498.7 + 103.1 | -356.7 (-585.3, -128.2) | **0.003** | -339.1 (-561.3, -117.0) | **0.003** |
| Decline in FEV_1_/FVC, %/y | -0.1 + 0.3 | -0.8 + 0.5 | -0.7 (-1.9, 0.5) | 0.230 | -0.8 (-2.5, 0.8) | 0.312 |

IOS, Impulse oscillometry; BDR, bronchodilator response; FEV_1_, forced expiratory volume in one second; FVC, forced vital capacity; COPD, Chronic Obstructive Pulmonary Disease.

* Logistic regression model; exacerbations at 2-year follow-up as binary variable 0 vs ≥ 1.

† Adjusted for age, sex, BMI, smoking status, pack-years, family history of respiratory diseases, occupation exposure, biomass exposure, history of asthma, exacerbations in previous year and baseline pre-bronchodilator FEV_1_.

‡linear mixed-effects model.

§ Adjusted for age, sex, BMI, smoking status, pack-years, family history of respiratory diseases, occupation exposure, biomass exposure, history of asthma and baseline lung function (FEV_1_, FVC, and FEV_1_/FVC)

¶ 2 pariticipants without Moderate to severe exacerbations data
